# External or internal fixation in the treatment of non-reducible distal radial fractures? A 5-year follow-up of a randomized study involving 50 patients

**DOI:** 10.3109/17453674.2011.618910

**Published:** 2011-11-24

**Authors:** Marcus Landgren, Daniel Jerrhag, Magnus Tägil, Philippe Kopylov, Mats Geijer, Antonio Abramo

**Affiliations:** ^1^Hand Unit, Department of Orthopedics, Clinical Sciences; ^2^Department of Hand Surgery Malmö/Lund; ^3^Center for Medical Imaging and Physiology, Lund University and Skåne University Hospital, Lund, Sweden

## Abstract

**Background and purpose:**

We have previously shown in a randomized study that in the first year after treatment, open reduction and internal fixation resulted in better grip strength and forearm rotation than closed reduction and bridging external fixation. In the present study, we investigated whether this difference persists over time.

**Patients and methods:**

The 50 patients included in the original study (mean age 53 years, 36 women) were sent a QuickDASH questionnaire and an invitation to a radiographic and clinical examination after a mean of 5 (3–7) years.

**Results:**

All 50 patients returned the QuickDASH questionnaire and 45 participated in the clinical and radiographic examination. In the internal fixation group, the grip strength was 95% (SD 12) of the uninjured side and in the external fixation group it was 90% (SD 21) of the uninjured side (p = 0.3). QuickDASH score, range of motion, and radiographic parameters were similar between the groups.

**Interpretation:**

The difference originally found between internal and external fixation in distal radial fractures at 1 year regarding grip strength and range of motion was found to diminish with time. At 5 years, both groups had approached normal values.

In unstable, non-reducible distal radial fractures, surgical treatment is recommended but can be complex. The choice of method is still controversial (Chen and Jupiter 2007), especially regarding the result over time (Downing and Karantana 2008). External fixation has been the method of choice for decades (Atroshi et al. 2006, Krukhaug et al. 2009), but with the introduction of the volar locking plate technique, internal fixation has rapidly become more and more popular—but without any solid evidence (Margaliot et al. 2005).

Recently, we showed in a randomized study that open reduction and internal fixation (O) of distal radial fractures using the TriMed fragment-specific system resulted in better grip strength and forearm rotation than closed reduction and bridging external fixation (C) (Abramo et al. 2009). The difference was seen early (at 3 months), which might be expected since the mobilization started earlier, but the difference prevailed at the 1-year follow-up. That study was initiated to investigate whether better anatomical reduction, achieved by the open technique, was important for the final result. Apart from the positive results regarding rotation and grip strength after internal fixation, we found a tendency for closed reduction to result in more malunions, but the difference was not statistically significant. Subjective outcome was similar between the groups, which we believe is either due to an absence of such a difference or to blunt outcome instruments. In the present report, we evaluated the same cohort at a later point in time, between 3 and 7 years, with the primary aim of determining whether the superior short-term results of internal fixation in unstable distal radial fractures persist over time.

## Methods

50 patients (mean age 48 (20–65) years, 36 women) with primarily irreducible, unstable, or comminuted distal radial fractures were randomized between May 2002 and December 2005 to be operated with either open surgery using the TriMed fragment-specific system (Figure) or closed surgery. The surgery was performed by 4 hand surgeons. The study was approved by the local research ethics committee (no. Lu 45/02).

**Figure:**
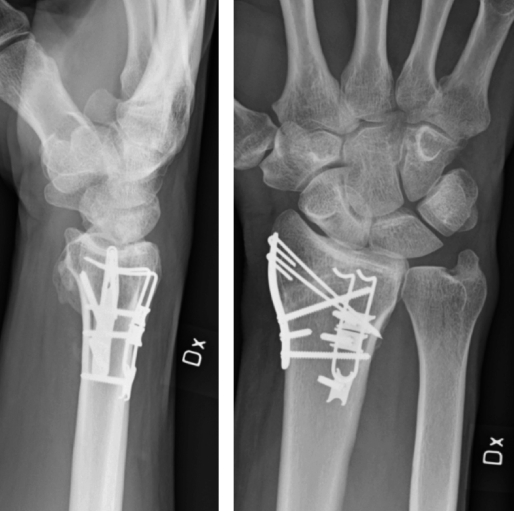
The TriMed fragment-specific system using radial pin plate, pins, and buttress pin.

### Clinical evaluation

The mean follow-up time was 5 (3–7) years. The subjective outcome was evaluated using the QuickDASH questionnaire (Beaton et al. 2005), a self-administered questionnaire consisting of 11 questions evaluating physical activities, severity of symptoms, and the effect of the injury on social activities. A score is calculated and converted to a scale from 0 to 100, with the higher score expressing the largest degree of disability. A validated Swedish version of the questionnaire was used (Gummesson et al. 2006). The general health of the patients was evaluated using the SF-36 questionnaire, which is constructed to survey health status in medical outcome studies. It consists of 8 scaled scores, which are the weighted sums of the questions in each section. Each scale is transformed into a 0–100 scale assuming that each question carries equal weight. A validated Swedish version of the questionnaire was used (Sullivan et al. 1995). The clinical examination was performed by two residents (ML and DJ). Grip strength (JAMAR) and range of motion (goniometer) were recorded.

### Radiographic evaluation

Lateral and anteroposterior radiographs were evaluated by a radiologist (MG). Standard measurements of radial inclination, ulnar variance, and dorsal angulation (Mann et al. 1992) were made using digital tools on the picture archiving and communication system (PACS) workstation. Secondary osteoarthritis—as indicated by reduced joint space width, subcortical sclerosis, subchondral cysts, and distal radioulnar (DRU) joint incongruence—was evaluated subjectively, and classified as being present or absent.

### Statistics

Student's t-test was used for continuous variables, such as range of motion (ROM) and grip strength. The radiographic results regarding DRU joint incongruence and the presence of osteoarthritis and reoperations were evaluated with Fisher's exact test. Wilcoxon rank sum test was used for QuickDASH score and SF36. We used SPSS software version 18.

## Results

50 patients filled out the QuickDASH questionnaire and 45 participated in the clinical examination. The QuickDASH score was median 9 (0–57) for the 45 patients who participated in the clinical examination and median 10 (0–34) for the 5 patients who did not attend the clinical examination (p = 0.8). Of the 26 patients in the O group, 1 patient had moved to another part of the country (QuickDASH score of 5) and 2 patients declined to participate (QuickDASH scores of 10 and 34). Of the 24 patients in the C group, 1 patient had a generalized neoplasm (QuickDASH score of 30) and 1 patient declined to participate (QuickDASH score of 0).

### Objective outcome

The mean grip strength was 31 kg (SD 13) in the O group and 30 kg (SD 11) in the C group (Table 1). The mean grip strength, given as a percentage of the uninjured side, was 95% (SD 12) in the O group and 90% (SD 21) in the C group (p = 0.3, 95% CI: –4 to 16 percent units). ROM was similar in the 2 groups (Table 1).

**Table 1.** Objective outcome in the open reduction and internal fixation group (O) and the closed reduction and bridging external fixation (C) group. Values are mean (SD)

	O (n = 23)	C (n = 22)	p-value
Grip strength			
% of uninjured side	95 (12)	90 (21)	0.3
kg	31 (13)	30 (11)	0.9
ROM (°)			
extension-flexion	126 (15)	119 (25)	0.3
pronation-supination	155 (17)	154 (20)	0.7
radial-ulnar	66 (16)	65 (13)	0.7

### Subjective outcome

The subjective outcome, measured as the QuickDASH score, was median 11 (0–46) in the O group and median 3 (0–57) in the C group (Table 3). In the O group, 4 patients had a QuickDASH score of 0 and in the C group 7 patients had a score of 0. In the C group, 3 patients scored over 30 in QuickDASH: one patient scored 36, one scored 48, and one scored 57. In the O group, the 4 patients who scored over 30 had scores of 34, 36, 45, and 45. General health—as measured with SF-36—was similar in the 2 groups.

### Radiographic outcome

The groups were similar regarding osteoarthritis, radial inclination, ulnar variance, dorsal angulation, and radial compression. 4 patients in the C group were classified as having radioulnar joint incongruency, as compared to 0 in the O group (p = 0.05) (Table 2).

**Table 2.** Radiographic outcome

	O (n = 23)	C (n = 22)	p-value
Radial inclination (°), mean (SD)	25 (4)	23 (5)	0.2
Ulnar variance (mm), mean (SD)	1.0 (2.1)	1.8 (2.1)	0.2
Dorsal angulation **^a^**, mean (SD)	–4 (7)	–2 (9)	0.5
Osteoarthritis (n)	2	4	0.4
DRU joint incongruence (n)	0	4	0.05

**^a^** Negative values indicate palmar angulation.

**Table 3.** Subjective outcome: QuickDASH and SF-36

	O	C	p-value
QuickDASH	15 (14) **^a^**	13 (16) **^a^**	0.6
	11 (0–46) **^b^**	3 (0–57) **^b^**	0.3
SF-36 **^b^**			
Physical functioning	90 (60–100)	100 (5–100)	1.0
Role-physical	100 (25–100)	100 (0–100)	0.4
Bodily pain	84 (22–100)	100 (12–100)	0.2
General health	82 (52–100)	84 (30–100)	0.9
Vitality	80 (35–100)	85 (20–100)	0.7
Social functioning	100 (25–100)	100 (12–100)	0.8
Role-emotional	100 (32–100)	100 (0–100)	0.3
Mental health	88 (32–100)	90 (16–100)	0.6

**^a^** Mean (SD)

**^b^** Median (range)

### Reoperations

6 fractures were reoperated due to symptomatic malunion, 1 in the O group and 5 in the C group. In addition, 3 patients in the C group were operated with carpal tunnel release and 2 for pin tract skin adherence. In the O group, 12 of the 26 patients had pins and plates removed after the fracture had healed, mainly due to radial nerve irritation. In 1 patient, a pin from the radial pin plate caused extensor tendon irritation to the fourth and fifth digit and a tenosynovectomy was performed (Table 4).

**Table 4.** Reoperations

	O (n = 26)	C (n = 24)	p-value
Reoperation due to malunion	1	5	0.09
Carpal tunnel release	0	3	0.1
Pin tract adherence	0	2	0.2
Osteosyntesis removal	12	0	< 0.001
Tenosynovectomy	1	0	0.5
Total	14	10	0.4

## Discussion

In the original 1-year follow-up of the present study, the mean grip strength was almost normalized in the O group (90%) but was lower (78%; p = 0.03, 95% CI: 2–21 percent units) in the C group (Abramo et al. 2009). In the present 5-year follow-up, both groups continued to improve and both approached the normal value of the uninjured side (95% for O group and 90% for C group; p = 0.3, 95% CI: –4 to 16 percent units). The statistically significant difference in grip strength between the 2 groups found at the 1-year follow-up had disappeared at the 5-year follow-up, and was smaller than the minimal clinically relevant difference chosen in the sample size estimation. However, the ratio between the injured and the uninjured sides was the same in the O group after 1 year as in the C group after 5 years (90%).

Also, the difference found in pronation-supination at 1 year had disappeared at 5 years, with almost normalized values in both groups. It may be speculated that the reason for this delayed normalization, in both rotation and grip strength, could still be a difference in the immediate postoperative protocol between the groups. The O group started mobilization at 2 weeks, as compared to 5 weeks in the C group. Regardless of choice of method, a continuous improvement, both in grip strength and forearm rotation, can still be expected even after the first postoperative year.

The subjective outcome, measured as DASH/QuickDASH, was similar between the groups—both early and late. In both groups, the DASH scores were low and similar between the 1- and 5-year follow-ups. The median QuickDASH score appeared lower in the C group, but the variance was greater. Of the 7 patients with high QuickDASH score after 1 year, 3 patients did not normalize and even increased in QuickDASH score at the late follow-up. The scores of 2 patients in the C group increased from 35 to 48 and from 34 to 57, and in 1 patient in the O group the score increased from 31 to 45. Reoperations due to removal of osteosynthesis material were more common in the O group (Table 4), and incongruency of the distal radioulnar joint was more common in the C group (Table 2). Osteoarthritis and malunion frequency was slightly higher in the C group, but not statistically significantly so; however, the study was not designed to find a difference in these parameters. There was no significant difference in QuickDASH score and ROM between the patients with and without malunion.

In the Cochrane review of surgical treatment of distal radial fractures, no evidence was found for the choice of a particular method or implant in the included 48 high-quality randomized studies (Handoll and Madhok 2003). There is some evidence to support the use of external fixation or percutaneous pinning, but “their precise role and methods are not established“ To our knowledge, there have been 8 randomized studies comparing open reduction and internal fixation to closed or indirect reduction (Kapoor et al. 2000, Grewal et al. 2005, Kreder et al. 2005, Leung et al. 2008, Abramo et al. 2009, Rozental et al. 2009, Wei et al. 2009, Xu et al. 2009). However, the results from older methods of internal fixation might differ from the results of the newer studies, and the comparisons may be irrelevant (Wei et al. 2009), due to the use of first-generation implants and/or lack of well-validated outcome instruments (Kapoor et al. 2000, Grewal et al. 2005).

To our knowledge, the present study is the second long-term follow-up (> 2 years) of a randomized comparison of distal radial fractures between internal fixation and external fixation (Kapoor et al. 2000, mean 4 years). We consider the 90% clinical follow-up acceptable and regarding the DASH/QuickDASH, 100% follow-up was met. The clinical examination was not performed blind because the scars were visible and showed what kind of operation had been performed. The subjective outcome in this study was measured with the short-form QuickDASH instead of the DASH questionnaire used in the first study, but the scores correlate well (Gummesson et al. 2006, Abramo et al. 2008).

In conclusion, internal fixation was better than external fixation regarding grip strength at 1 year, but at the 5-year follow-up both groups had approached normal values.
